# Haptenation: Chemical Reactivity and Protein Binding

**DOI:** 10.1155/2011/839682

**Published:** 2011-06-30

**Authors:** Itai Chipinda, Justin M. Hettick, Paul D. Siegel

**Affiliations:** Health Effects Laboratory Division, National Institute for Occupational Safety and Health, Centers for Disease Control and Prevention, MS L-2040, 1095 Willowdale Road, Morgantown, WV 26505, USA

## Abstract

Low molecular weight chemical (LMW) allergens are commonly referred to as haptens. Haptens must complex with proteins to be recognized by the immune system. The majority of occupationally related haptens are reactive, electrophilic chemicals, or are metabolized to reactive metabolites that form covalent bonds with nucleophilic centers on proteins. Nonelectrophilic protein binding may occur through disulfide exchange, coordinate covalent binding onto metal ions on metalloproteins or of metal allergens, themselves, to the major histocompatibility complex. Recent chemical reactivity kinetic studies suggest that the rate of protein binding is a major determinant of allergenic potency; however, electrophilic strength does not seem to predict the ability of a hapten to skew the response between Th1 and Th2. Modern proteomic mass spectrometry methods that allow detailed delineation of potential differences in protein binding sites may be valuable in predicting if a chemical will stimulate an immediate or delayed hypersensitivity. Chemical aspects related to both reactivity and protein-specific binding are discussed.

## 1. Introduction

The term, “hapten,” was coined by Landsteiner and Jacobs [1] and is derived from the Greek “hapten”, meaning “to fasten.” Haptens are low molecular weight (LMW; <1000 daltons) chemicals that must bind to a carrier molecule to be antigenic. The carrier is usually an endogenous or exogenous protein to which the LMW chemical is covalently bound. The hapten hypothesis was originally proposed to explain both humoral and cellular immune responses to LMW chemicals observed by Landsteiner and Jacobs [1] in their research. The absolute requirement for covalent binding of a hapten to a protein for immune recognition in the development of all drug/LMW chemical allergies has been challenged in recent years [2], but substantial evidence exists for this to be a prominent mechanism through which chemicals and drugs or their metabolites become antigenic.

The role of chemical reactivity has been proposed to be one of the major determinants in allergic contact dermatitis (ACD). Over the years, extensive databases containing representative chemicals that are skin sensitizers have been published [3–5]. In the context of occupational health, predictive toxicology, and ensuring overall safety of manufactured products, it is important that skin sensitization potential of new and existing chemicals be assessed. The use of guinea pigs has been the experimental model of choice in evaluating the skin sensitization potential of chemicals [6, 7] until about a decade ago when the local lymph node assay (LLNA) was adopted after extensive interlaboratory validation [8]. 

Although ACD is a Type IV hypersensitivity response, the ability of a chemical to bind to macromolecules is also thought to be important for immediate (Type I) hypersensitivity sensitization and reactions in both the skin and lung. Presently, why a specific LMW chemical predominately skews the immune system toward Type I versus Type IV hypersensitivity is not known. Electrophilic reactivity alone does not seem to distinguish respiratory and dermal sensitizers such as toluene diisocyanate and dichlorobenzene, respectively. Selective protein targets or sites on a protein may be important and recent advances in protein mass spectrometric analysis now provide the capability to better explore how and where such chemicals bind. The present paper discusses these physical chemical aspects related to formation of the hapten-carrier protein complex.

## 2. Electrophiles and Reactivity

The hapten hypothesis was developed from the interaction of nucleophilic moieties on proteins with chemicals that are electrophilic. Adduct formation has been demonstrated to be more feasible with electrophilic chemicals [9]. In the following description, haptenation within the skin is discussed, as most of the research knowledge gained has been through examining the relationship between chemical reactivity and allergenicity in this organ system. An analysis of two published databases [4, 5] containing more than 300 chemicals demonstrated to be allergens by the LLNA reveals that approximately 40% of the skin sensitizers have at least an electrophilic center that is amenable to nucleophilic attack. From an organic chemistry perspective, formation of such adducts is via covalent bonds and to a certain extent coordination bonds. This is chiefly because covalent and coordination bonds have bond energies ranging from 200 to 420 kJ/mol compared to hydrophobic, dipolar, and ionic interactions with bond energies <50 kJ/mol. The high bond energies enable covalent adducts to survive the intracellular antigen processing of the haptenated protein into short peptides for cell surface expression by MHC complexes. 

Both guinea pig ACD models and LLNA data have been used to develop a number of structural-activity-relationship models relating chemical reactivity and hydrophobicity to skin sensitization potency [10]. Hapten reactivity data generated from our laboratory [11] supports a central role for chemical reactivity in allergic sensitization. Reactivity rate constants (*k*) were obtained for twenty five electrophilic haptens using a thiol-based probe, 4-nitrobenzenethiol (NBT). This *k* is a measure of the speed at which an electrophilic hapten will bind to a nucleophilic center. A very high correlation was obtained between allergic potency (EC3) of electrophilic haptens as determined by the LLNA and the reactivity rate constants (*r* ^2^ = 0.74, independent of reactivity domain). More recently, we have developed a similar assay using an amine-based probe, and preliminary results suggest that a similar relationship between EC3 and reactivity with a nucleophilic amine exists [12]. 

Employing chemical reactivity as an endpoint to probe target toxicity is neither restricted to skin sensitization nor is it a new concept. For example, the ability of a chemical to react with biomolecules has been used as a predictor for aquatic toxicity [13, 14] and carcinogenesis [15]. Landsteiner and Jacobs [1] noted correlations between a chemical's ability to cause skin sensitization in guinea pigs with its reactivity to aniline [1]. Interestingly, Landsteiner and Jacobs optimized their chemical reactions by altering pH or elevating the reaction temperature to observe reaction to nucleophiles like butylamine and aniline. It has to be noted that many covalent adducts that would otherwise form under harsh conditions are not formed as reaction conditions (i.e., temperature, pH) and medium are adjusted to physiological-like conditions. More recently, reactivity assays utilizing cysteine, lysine, glutathione, and several model peptides have been developed and efforts are underway to validate them as alternative *in chemico* methods for screening skin sensitizers [16–19]. The underlying concept for all these assays is electrophile-nucleophile interaction. 

An understanding of the chemistry of electrophiles is required to produce qualitative and quantitative data and also for determination of an appropriate solvent system for reactivity assessment. For example, cinnamic aldehyde (EC3 = 3; [20]) and bromohexadecane (EC3 = 1.75; [21]) which are both moderate sensitizers in the LLNA require different solvent systems (*in vivo, in vitro, and in chemico)* for assessment of allergenicity. While all chemicals with reactive electrophilic centers will form covalent adducts with proteins, mechanistic pathways are different for different chemicals and may determine the type and strength of adduct formed.  Table 1 illustrates the five common mechanistic domains and the electrophilic centers amenable to nucleophilic attack. An extensive analysis of mechanistic domains, their subcategories, and “special cases (domain not clearly defined or >1 domain for a single chemical)” has been discussed in recent reviews [9, 22, 23]. Mechanistic domains for protein interaction should not be confused with chemical classes, which are structural-based classifications. Mechanistic domains are functional reaction groups that are targeted as alerting pointers to a chemical's possible reaction with a protein and thus classify chemicals based on their reaction chemistry. For example, benzoquinone and 2,3-butanedione are both ketones but they belong to the Michael acceptor (MA) and Schiff base former (SBF) mechanistic domains, respectively. 

Use of chemical classes instead of mechanistic domains has been noted to have a number of limitations. The relative alkylation index (RAI) of Roberts and Williams was developed using chemical classes and could only predict reactions of a select group of chemical classes [24]. When the RAI was modified to predict protein haptenation based on mechanistic domains, it attained applicability to a wider range of chemicals as discussed by Patlewicz et al. [10]. Our initial experience with 19 chemicals spanning three mechanistic domains (MA, S_N_1/S_N_2, AA) suggests that restricting correlations between *k* and LLNA data to mechanistic domains was not necessary to provide a good prediction of an electrophilic hapten's allergenic potency [11]. Despite a good prediction of allergenic potency that was obtained across all binding mechanisms, separation of allergens by mechanistic domain improved the correlations from linear regression analysis. A further comparison of MA and S_N_1/S_N_2 to EC3 by regression analysis reveals similar slopes, but different Y-intercepts (*P* = .006), confirming that the mechanism of electrophilic binding does influence allergenic potency.

## 3. Hapten Bioavailability and Reactivity Methods Development

Another major physical chemical consideration involves the bioavailability of the hapten. For skin, this is penetration of the chemical across the stratum corneum and is thought to be mainly a function of the chemical's solubility (octanol/water partitioning; log  *P*). Models for prediction of skin sensitization take this into consideration and usually include both reactivity and hydrophobicity parameters. While the inclusion of penetration rate may be ideal for accurate prediction of skin sensitization potential, in practice, it is not a significant parameter [11, 25] as hapten reactivity rates alone highly correlate with LLNA potency. Most electrophilic haptens tested in our model fall into the desired range of log  *P* values (−1.4 to 4) for skin absorption and lipophilicity. It is possible that bioavailability may exert a greater influence on allergenic potency for extremely hydrophilic and lipophilic haptens. Roberts and Natsch [26] included both reactivity and hydrophobicity parameters in their modeling of allergenic potency and noted that the influence of reactivity was greater than that of solubility for predicting allergenic potency. 

Methods for the assessment of electrophilic chemical allergen binding to (protein) nucleophiles developed in recent years have been primarily non-kinetic-based assays that measure the loss of the unconjugated nucleophile. Development of these methods is based on the assumption that hapten bioavailability for chemicals with log  *P* values between −1.4 and 4 is approximately the same. The nucleophilic probes reported include glutathione [16, 18, 27] or model peptides with a free cysteine thiol or lysine amine [17, 28]. These assays report percent depletion as the reactivity index for a given chemical. The rationale for design of a particular synthetic peptide, including the choice of neighboring amino acids in the hepta-peptides and why only seven amino-acid peptides are used, has not been delineated for most of the peptide probes proposed. The exception is the peptide, Ac-NKKCDLF (Cor1-C420) [29], derived from AA417-423 of the human Coronin 1 protein, where the Cys420 is thought to be highly reactive to electrophiles [30]. The original HPLC-based peptide reactivity assay has since had numerous modifications and improvements including the inclusion of LC-MS to characterize the adducts [29, 31] and configuring it to high throughput kinetic profiling for more accurate determination of rate constants [26]. The modifications seek to interrogate the chemistry behind the peptide depletion by the electrophilic skin sensitizers and also begin to move towards high throughput assay development. The generation of reaction kinetics data was another important aspect that had been lacking in the original peptide reactivity assay. Data on peptide depletion based on varying initial electrophile concentrations [26] results in more accurate determination of reactivity constants as opposed to derivation of the RC50 (2 h assay) [18, 27] and peptide depletion (24 h assay) [32] as reactivity indices. These endpoint determinations do not adequately capture the nature of the chemical kinetics involved in these electrophile-nucleophile interactions. The fact that values are measured at fixed time points under pseudo-first-order conditions (electrophile ≫ peptide) is a limitation of these assays. Fixed time points of several hours do not take into consideration the initial reaction and chemical kinetics involved which have a bearing on whether the reaction with the peptide is going to be linear or not throughout its duration. 

The high throughput kinetic profiling (HTKP) method [26] was able to address some of the shortcomings of earlier reactivity assays with respect to reaction times and the chemistry of electrophiles that do not adhere to pseudo-first order kinetics. Measurements of peptide depletion/reactivity were done at several time points for varying initial concentrations of the sensitizers, and compensations were made for the “drowning out effects” [26] and loss of test chemicals due to evaporation [26]. While this presented a breakthrough in terms of determining more accurate rate constants that could be tied to LLNA potency of the electrophiles, determining rates of rapidly reacting sensitizers such as benzoquinone and nitrobenzyl bromide still presented challenges and the rate constants for these sensitizers had to be estimated rather than measured. The application of stopped flow techniques [11] to measure the rate constants of these rapid electrophile-nucleophile interactions introduced a novel chemoassay that was superior with respect to the detectable range of electrophilic reactivity. Other confounders such as potential loss of nucleophile due to evaporation and even oxidation were eliminated ensuring the measurement of reaction rates from solution kinetics. Using this technique, the modeling and correlations of reactivity constants to LLNA data were able to utilize measured rate constants instead of estimates.

Our current method utilizes the depletion of NBT with the assumption that the electrophile-nucleophile reactions are characterized by adduct formation. Peptide binding studies [31], which have included characterization of the chemical products formed, have noted oxidation of the peptide thiol producing an apparent loss of parent peptide whenever there was absence of adduct formation. The buffered organic media precluded oxidation of nucleophile to species other than the disulfides of which the rate would have been slower than the electrophile-nucleophile reaction. We are currently evaluating an amine-based probe, pyridoxylamine (PDA) that will better assess amine-selective electrophiles. This assay, which captures adduct formation without interference from side reactions like oxidations, might be of greater utility than the previously reported assays where complications arose from side reactions. Several advantages in screening for hapten potency by binding to NBT and PDA include the ability to quickly obtain both initial and overall binding rates for both extremely fast or slow reactions, increased accuracy of rate constants, analysis which can be conducted on relatively inexpensive stopped-flow spectrophotometers, and the ability to model potential reaction mechanisms, competing mechanisms, and intermediate products.

## 4. Nonelectrophilic Haptens

The allergenicity of nonelectrophilic compounds and metals, which have not been shown to be metabolically bioactivated cannot be explained by direct electrophile-nucleophile interaction chemistry. When the potential for chemicals to induce mutations in *Salmonella* was used as a surrogate for electrophilicity [33], correlation of electrophilicity with occurrence of ACD in humans from 355 randomly chosen chemical allergens demonstrated that only 30%–40% of the contact allergens were electrophilic. Nonelectrophilic compounds have been studied including thiols such as the rubber accelerator allergens, mercaptobenzothiazole (MBT), mercaptobenzothiazole disulfide (MBTS), zinc diethyldithiocarbamate (ZDEC), and tetraethylthiuram disulfide (TETD). Metabolic activation to electrophilic metabolites may account for the potency of a portion of these allergens; however, haptenation mechanisms other than electrophile-nucleophile interactions have been proposed. Guinea pig studies with MBT and several structural analogues demonstrated that sensitization was most likely a result of protein haptenation via disulfide formation [34]. ACD cases which have also been reported for chemicals like diallyl disulfide [35] and lipoic acid [36] which are thiols further indicate that disulfide formation may be a common mechanism for chemical thiol haptenation. The conclusion that MBT/MBTS haptenates proteins via disulfide formation was supported by enzyme inhibition and protein-binding studies where binding of MBTS to enzymes (reductases) and other protein cysteine residues was through disulfide formation [37]. Bioactivation of MBT, which could potentially lead to an electrophilic hapten was not observed [37], suggesting that MBT is not a metabolically activated prohapten. To date, there are no reports of MBT bioactivation by cutaneous cytochrome P450s (CYPs) enzymes. 

TETD has also been shown to haptenate proteins through the formation of disulfide linkages [38]. As a strong ligand, TETD binds coordinatively to metalloproteins, the mechanism by which it inactivates aldehyde dehydrogenase [39] and anhydrases [40]. Whether the same properties (binding and inactivating dehydrogenase) that make TETD a suitable alcohol abuse deterrent apply to skin sensitization biology is yet to be determined. If accessible, chemicals like TETD and ZDEC (through transmetallation) will chelate the metal ions in a porphyrin center. Absorbance measurements, dialysis experiments, and mass spectrometry after haptenation of zinc/copper-superoxide dismutase (SOD) with ZDEC indicated that the DEC from ZDEC were strongly chelated to the copper ion on SOD [38, 41, 42]. The lack of binding between ZDEC and the apoenzyme was confirmatory of the chelation chemistry being the probable mechanism of haptenation [38]. It has to be noted though, that ZDEC and TETD, unlike MBT, can potentially be metabolized to electrophilic species, through sulfoxidation [43], which would then haptenate proteins through electrophile-nucleophile interactions. Human cytochrome P450 enzyme that can metabolize the thiocarbamates has been identified [43]. Contact allergens may also undergo nonenzyme catalyzed, air oxidation to electrophilic intermediates. Lepoittevin [44] suggested the separate classification term, prehapten, to refer to chemicals that undergo nonenzymatic transformation to the active form.

## 5. Metal Allergens

The formation of coordinate bonds has been touted as the mechanism behind metal ion-induced allergies. Commonly encountered metal allergens are transition and trace metals which include nickel, cobalt, chromium, beryllium, platinum, and gold [45]. Binding of metals to proteins stems from the polarized nature of the metal atoms which allows them to accept electrons from electron rich ligands. Metals are capable of forming geometrically, highly defined coordination complexes with four or six electron donors. The electron donors are mainly nitrogen or oxygen in amino acid side chains of appropriate proteins or peptides [46]. The binding of nickel to albumins was shown to have the capacity to stimulate Ni-reactive T cells in the presence of appropriate antigen presenting cells (APC) [47–49]. The comparable response of T cells to determinants formed by hapten peptides in a major histocompatibility complex- (MHC-) binding groove versus the Ni–MHC–peptide complexes strongly suggested that the coordinate binding is a feasible mechanism for metal-induced allergies [50]. Thus metals represent nonclassical haptens in the sense that coordinate bonds which form metal-protein complexes are not sufficiently strong to survive antigen processing that classical haptens undergo. The binding of metals like Ni to cell surface proteins like MHC would indicate a more plausible mechanism as it bypasses intracellular antigen processing steps. This type of protein binding would suggest that sensitization to metals is protein independent as long as the cell surface protein is able to chelate the metal and present it to T cells. This protein/peptide independence can also be attributed to the observed cross-reactivity between different metal ions [51, 52] and in the case of Ni it was proposed that Ni may link T cell receptors (TCR) and MHC in a peptide-independent manner [51]. Another mechanism that has been postulated for metals, Ni in particular, is binding to specific carrier proteins that ensure survival of the critical Ni-peptide complex throughout the transport and processing through the epidermis and dermis, and then transfer of the metal to short-lived, high-affinity coordination sites created within certain TCR–MHC contact zones [49].

## 6. Pharmacological Interaction Mechanism

Additional allergenic compounds that are otherwise chemically inert (unable to directly haptenate proteins) have been shown to cause lymphocyte proliferation [53] suggesting a different mechanism by which they are able to stimulate TCR. The chemicals, which are usually drugs associated with adverse hypersensitivity reactions, have been shown to bind to the MHC on the basis of their conformation rather than reactivity. This kind of binding, referred to as pharmacological interaction (p-i), is labile and more effective when it is on the MHC and within proximity of the TCR [53]. Experiments that involved washing steps after binding of the MHC proteins and before stimulation of T cells resulted in lack of stimulation indicating that the weak protein binding is reversible. Washing was ineffective with chemicals that were covalently bound to MHC peptides. Other evidence supporting the model for direct interaction of the sensitizer with both TCR and the MHC includes the kinetics of T-cell activation which happens much faster than would be feasible if antigen processing was occurring. The p-i concept has been used to explain hypersensitivity of drugs such as lidocaine, sulfamethoxazole, mepivacaine, celecoxib, carbamazepin, lamotrigine, and ciprofloxacin [54–58] which are not haptens/prohaptens but still elicit an immune response because their conformation allows them to fit into the MHC-TCR sandwich. The chemical p-phenylenediamine (PPD) has been shown to stimulate TCR via this model in addition to its ability to haptenate proteins [59]. The large number of TCR available (>10^12^) [53] makes it plausible that some chemicals will have conformations that allow them to associate with the TCRs.

## 7. Prohaptens

Prohaptens are chemicals that are not protein reactive unless they are metabolically activated to electrophilic species. It has also been proposed that prohapten chemicals that undergo air oxidation to reactive species be classified separately as prehaptens, but the criteria on when a chemical is a pro- or prehapten is confusing [44]. Many chemicals such as PPD, isoeugenol, and limonene are assigned to the pro- or prehapten category on evidence indicating that they are either metabolized or undergo air oxidation. With an estimated one third of known skin sensitizers needing metabolic or abiotic activation to react with skin proteins [60], it is important that a clear distinction be made between haptens and prohaptens. The recent emphasis on alternative screening methods to avoid animal use for screening of both contact and respiratory allergens depends on identification of prohapten mechanisms to minimize false negative classification of sensitizing chemicals. While guinea pig tests and the LLNA are able to identify prohaptens, nonanimal assays have to include a metabolizing system to bioactivate these otherwise nonsensitizing chemicals. With animal-based assays, the lack of false negatives with prohaptens attests to the fact that the skin is an important site of metabolizing enzymes even though its metabolic capability has not been fully characterized [61]. Bioactivation of prohaptens commonly involves oxidative processes, with the cytochrome P450 system (CYPs) playing a major role in the biotransformation of the majority of prohaptens to sensitizers. CYP enzymes that have been detected at the mRNA level in the skin include CYP1A1, 1B1, 2B6, 2E1, and 3A5 [62]. While the proteins were not shown, this mRNA expression was consistent with expression levels that were found for normal human epidermal keratinocytes (NHEKs) and dermal fibroblasts [63–65]. Other metabolic enzymes identified in the skin include monooxygenases, dehydrogenases, esterases, amidases, and Phase II enzymes which are mainly transferases [66, 67]. Keratinocytes have also been shown to possess prohapten metabolizing capacity [63, 68]. To date, there have been few studies on the characterization of prohapten bioactivation by CYP enzymes. Prohaptens that have been studied include the polyaromatic hydrocarbons (PAHs), cinnamic alcohol, carvone oxime, isoeugenol, and diphenylthiourea [62, 69–72]. Once bioactivated to an electrophilic species, haptenation of proteins proceeds via one of the previously discussed mechanisms (Table 1). 

Metabolic activation has so far proven to be the Achilles' heel for many *in vitro* and *in chemico *assays. The need to exhaustively interrogate the use of metabolic systems where prohaptens are concerned has been discussed in detail [73]. Recent *in vitro* studies [62, 74, 75] have included metabolic systems to detect prohaptens with marked success, but more work needs to be done to identify metabolic systems that are more representative of skin metabolism.

## 8. Protein-Selective Haptenation Targets: The Diisocyanate-Albumin Example

Exposure to diisocyanates in the workplace is one of the leading causes of occupational asthma. It is hypothesized that isocyanate acts as a hapten by reacting with protein carriers via nucleophilic attack; however, the ultimate form of these protein-isocyanate conjugates that functions as allergens *in vivo* is, as yet, unknown. The diverse functional groups present in proteins (amines, amides, thiols, alcohols, carboxylic acids) present a large number of potential reaction sites for the diisocyanate (dNCO). However, previous studies have suggested that under physiological conditions, these are limited to N-terminal *α*-amines, the sulfhydryl group of cysteine, the hydroxyl groups of serine and tyrosine, the *ε*-amine of lysine, and the secondary amine of the imidazole ring of histidine [76]. Understanding the products formed by reaction of allergenic dNCOs such as methylene diphenyldiisocyanate (MDI) and toluene diisocyanate (TDI) with biological molecules is critical to understanding the mechanisms by which these chemicals affect living systems. Tandem mass spectrometry performed on a quadrupole time-of-flight (qTOF) mass spectrometer [77] is particularly well suited for the characterization of chemically modified proteins. Proteins of interest may be digested with a proteolytic enzyme (such as trypsin) and the resultant peptides analyzed with high sensitivity and mass accuracy. Because covalent modification of an amino acid residue results in a change in that residue's mass, accurate mass determination of the fragment ions produced by collision-induced dissociation [78] allows unambiguous assignment of the site of modification. Such experiments have become routine for the analysis of posttranslational modifications such as acetylation, glycosylation, and phosphorylation, among many others [79]. 

Recent efforts in our laboratory [81, 90] and others [80] have begun to focus on harnessing the power of tandem mass spectrometry to determine how and where dNCOs modify model peptides and proteins. Hydrolysis of the isocyanate functional group to a primary amine is a competing reaction under aqueous conditions. These hydrolyzed isocyanate amines may then undergo nucleophilic addition to another dNCO molecule. Therefore, conjugation products observed upon reaction of dNCOs with model peptides and proteins *in vitro* results in a complex variety of different reaction products, including intra- and intermolecular crosslinking, dNCO self-polymerization, and dNCO hydrolysis. Our initial study focused on determining the site of dNCO modification on model bioactive peptides conjugated under aqueous conditions [81]. Analysis of these conjugates by tandem mass spectrometry revealed that the dNCO was bound preferentially to the N-terminal amine of each of the peptides examined. Furthermore, when a peptide with an N-terminal residue containing a side chain amine (lysine, arginine) was reacted with dNCO, intramolecular crosslinking with the side chain amine becomes competitive with hydrolysis, however, the reactivity decreases as the residue is displaced further from the N-terminus. The results of this peptide study suggested agreement with a long-held hypothesis that the N-terminal amine of protein chains is a likely target for isocyanate conjugation [76, 82]. Studies of the kinetics of isocyanate binding with protein functional groups [83] determined that at pH 7, reaction with an N-terminal amine should proceed approximately 100 times faster than the *ε*-amine of the lysine side chain. The difference is due to the relative pK_a_ of the two functional groups (*α*-NH_3_^+^ pK_a_~ 9 versus Lys *ε*-NH_3_^+^ pK_a_~10.5), as dNCO conjugation proceeds through the neutral –NH_2_ rather than the charged –NH_3_^+^ species.

Although tandem MS studies of dNCO-conjugated peptides are useful for determining the diverse chemical species produced when dNCO binds amino acids, they do not produce the unique chemical microenvironments presented by the complex three-dimensional structure of proteins. In order to understand how dNCOs react in these complex environments, studies on model proteins are essential. Serum albumin is an appropriate model protein, as it is monomeric and its sequence and three dimensional structure have been well defined. It is naturally abundant (35–50 mg/mL in serum), found in most tissues, and has been identified as a target of dNCO binding *in vivo *[84–89]. Recently, our laboratory completed an extensive analysis of the binding sites of TDI on human serum albumin [90]. At high (40 : 1 dNCO : protein) ratios, near-stoichiometric binding was observed; TDI binds at thirty-seven sites on the protein, including the N-terminal amine on aspartic acid at position one and the side chain of thirty-four lysine residues. At lower conjugation ratios (1 : 2 dNCO : protein), a small subset of these thirty-seven sites is conserved, with binding observed at the N-terminus and four lysine residues, suggesting these sites are preferred binding sites. Kristiansson and coworkers [91] determined that at a tenfold molar excess, HHPA bound to thirty-seven sites on human serum albumin, including the N-terminal aspartic acid and thirty-six lysine residues.

**Figure 1:**
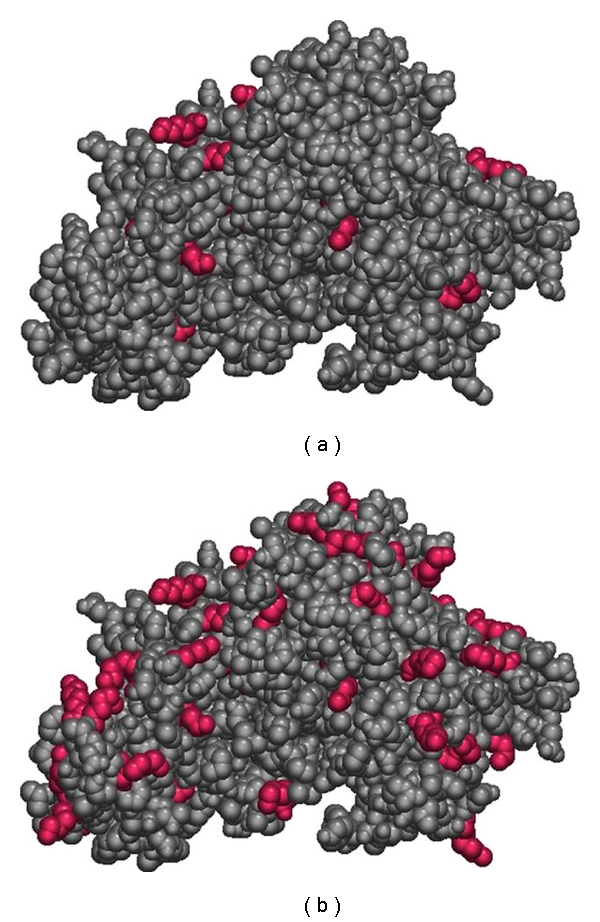
(a) Lysine residues observed bound to MDI and TDI on human serum albumin. (b) All lysine residues on human serum albumin.

Interestingly, all 59 lysine residues of human serum albumin have been determined to be solvent accessible, but only 37 are reactive toward TDI, while 19 are reactive toward MDI (Hettick and Siegel, unpublished data). Human serum albumin is a highly charged molecule, in part accounting for its high solubility. For example, Figure 1 provides two views of human serum albumin based on its crystal structure [92]. In Figure 1(a), the lysine residues that bind MDI and TDI are highlighted, whereas in Figure 1(b), all 59 lysine residues are highlighted. Steric effects are insufficient to explain the difference between accessibility and observed binding. It is therefore likely that the microenvironment of the binding site(s) determines whether or not a certain lysine residue is reactive toward isocyanate. Lysine 199, which is known to bind hydrophobic anions such as aspirin and benzyl penicillin, was also determined to be a predominant binding site for both TDI and MDI. Gerig and Reinheimer [93] determined that the pK_a_ of the aspirin binding site (later determined to be Lys199) of albumin was 7.9. These authors hypothesized based on the reactivity of human serum albumin with dinitrofluorobenzene that there exist two lysines on HSA that have a pK_a_ as low as 7.9. In addition, Lys199 has been shown by molecular dynamics calculations to be predominantly uncharged, undergoing proton transfer with the nearby Lys195 [94]. This study elegantly suggests the reason we observed dNCO bound to Lys199, and not the nearby the Lys195. Other sites noted to be abundant binders of dNCO, such as Lys439 and Lys525, have been observed to undergo nonenzymatic glycosylation *in vivo*. Glycosylation is generally observed to occur at lysine residues located near another amino group, presumed to be charged [95]. Lys439 is located in a region with two other nearby lysine residues (Lys432 and Lys436) and Lys525 is part of a dilysine motif. 

**Figure 2:**
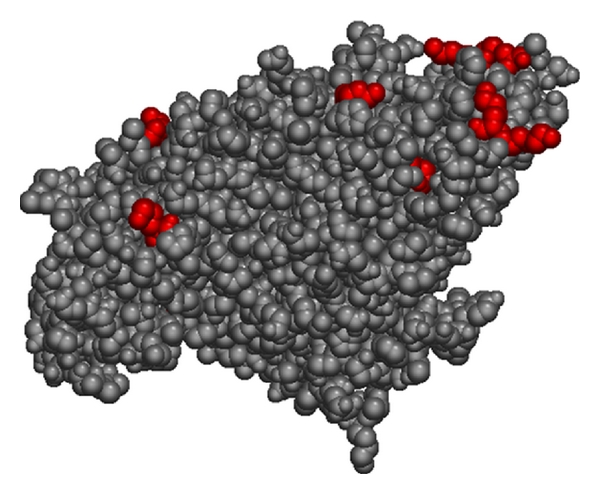
Lysine residues reactive to TDI but not MDI.

More recently, we have begun an investigation to compare the binding sites of TDI and MDI on serum albumin under identical conditions (40 : 1 dNCO : HSA; 50 mM NH_4_HCO_3_, pH 7.9). Under these conditions, MDI is observed to bind to a subset of 19 of the 37 sites observed for TDI (see Table 2). Although it would be tempting to attribute differences in binding between TDI and MDI to steric effects, as seen in Figure 2, many of the lysine residues that are reactive toward TDI but not MDI are open and highly accessible. We therefore hypothesize that the difference in observed binding between TDI and MDI is attributable to a combination of steric effects and the increased reactivity of TDI. The electron withdrawing character of the second N=C=O group on the aromatic ring of TDI significantly increases the reactivity of the first isocyanate. In contrast, the reactivity of the isocyanate functional group(s) on MDI is lower because the p-ethyl phenylisocyanate substituent is less electron-withdrawing. Wisnewski and coworkers also examined the reaction products between MDI and human serum albumin by HPLC-MS/MS. Their data indicated 14 binding sites on albumin, including 12 lysine and 2 asparagine residues, in relatively good agreement with the results presented in Table 2. In addition, these authors suggested that the four “dilysine” (KK) motifs in human serum albumin are important binding sites, and that MDI shows reactive specificity for the second lysine. As discussed previously, the ability of a lysine residue to transfer its proton to a nearby lysine or histidine residue may, in fact, lead to increased reactivity toward dNCOs. However, as dilysine motifs account for 8 of 36 TDI binding sites and 6 of 19 MDI binding sites, it is clear that two lysine residues in a “KK” arrangement are not essential to binding.

## 9. Conclusion

It has been recognized for over 80 years that the ability of a hapten to react to a protein was central to its ability to produce allergic sensitization. Haptenation of a protein can occur by multiple mechanisms (primarily electrophilic attack) and is dependent on many factors such as chemical properties, bioavailability, and site of exposure. Recent studies have greatly expanded the knowledge in this area by demonstrating that it is the rate at which an electrophilic hapten reacts with a nucleophile center that is a central determinant in its dermal sensitization potency. In addition, the chemical mechanism of binding (i.e., mechanistic domain) also influences allergenic potency. These physical chemical factors, however, have not been shown to be related to skewing of the immunological response toward Th1 versus Th2. Studies are currently utilizing modern proteomic mass spectrometry to identify hapten binding sites on proteins and to identify specific hapten target proteins. It is possible that the protein-specific factors may play a role in the ultimate nature of the immune response.

## Figures and Tables

**Table 1:** Common mechanistic domains.

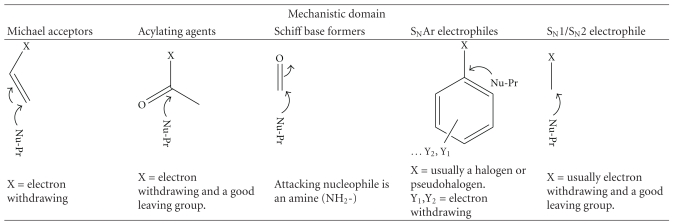

**Table 2:** 

Residue	MDI	TDI
Asp1*	X	X
Lys4*	X	X
Lys12	X	X
Lys73	X	X
Gln104		X
Lys106		X
Lys136	X	X
Lys137*	X	X
Lys159		X
Lys190	X	X
Gln196		X
Lys199*	X	X
Lys205		X
Lys212		X
Lys262*		X
Lys274		X
Lys276		X
Lys281		X
Lys351*	X	X
Lys378		X
Lys402		X
Lys413*	X	X
Lys414*	X	X
Lys432*	X	X
Lys436*	X	X
Lys439*	X	X
Lys444*	X	X
Lys524	X	X
Lys525*	X	X
Lys534		X
Lys536	X	X
Lys541*	X	X
Lys545		X
Lys557		X
Lys560		X
Lys573		X
Lys574		X

*MDI binding sites indentified in [80].

## Abbreviations

AA: Acylating agents
ACD: Allergic contact dermatitis
CYPs: Cytochrome P450s
dNCO: Diisocyanate
EC3: Concentration of allergen producing a 3-fold lymph node cell proliferation = LLNA threshold
HPLC-MS/MS: High performance liquid chromatography-tandem mass spectrometry
HSA: Human serum albumin
LLNA: Murine local lymph node assay
LMW: Low molecular weight
log *P*: Octanol/water partitioning
MA: Michael acceptor
MHC: Major histocompatibility complex
MBT: Mercaptobenzothiazole
MBTS: Mercaptobenzothiazole disulfide
NBT: 4-nitrobenzenethiol
MDI: Methylene diphenyldiisocyanate
PPD: p-phenylenediamine
qTOF: Quadrupole time-of-flight
RAI: Relative alkylation index
SBF: Schiff base former
S_N_1/S_N_2: Nucleophilic substitution unimolecular or bimolecular
SOD: Superoxide dismutase
TCR: T-cell receptors
TETD: Tetraethylthiuram disulfide
TDI: Toluene diisocyanate
ZDEC: Zinc diethyldithiocarbamate.
